# Interhospital transmission of vancomycin-resistant *Enterococcus faecium* in Aomori, Japan

**DOI:** 10.1186/s13756-022-01136-5

**Published:** 2022-07-23

**Authors:** Norihiro Saito, Junichi Kitazawa, Hiroko Horiuchi, Takeo Yamamoto, Masahiko Kimura, Fumio Inoue, Mika Matsui, Satoko Minakawa, Masamichi Itoga, Junichiro Tsuchiya, Satowa Suzuki, Junzo Hisatsune, Yoshiaki Gu, Motoyuki Sugai, Hiroyuki Kayaba

**Affiliations:** 1 Department of Clinical Laboratory Medicine, Hirosaki University Graduate School of Medicine, 5 Zaifucho, Hirosaki, Aomori 036-8562 Japan; 2 Clinical Laboratory, Hirosaki University Hospital, Hirosaki, Japan; 3 Aomori Infection Control Network, Hirosaki, Japan; 4 Infection Control Office, Division of Clinical Laboratory, Aomori Prefectural Central Hospital, Aomori, Japan; 5 Clinical Laboratory, Hachinohe City Hospital, Hachinohe, Japan; 6 Clinical Laboratory, Hachinohe Red Cross Society Hospital, Hachinohe, Japan; 7 Clinical Laboratory, Hirosaki National Hospital, Hirosaki, Japan; 8 Antimicrobial Resistance Research Center, National Institute of Infectious Diseases, Tokyo, Japan; 9 AMR Clinical Reference Center, National Center for Global Health and Medicine, Tokyo, Japan

**Keywords:** Vancomycin-resistant *Enterococcus* (VRE), Outbreak, Interhospital transmission, Infection control

## Abstract

**Background:**

Spread of vancomycin-resistant *Enterococcus* (VRE) is a global concern as a significant cause of healthcare-associated infections. A series of VR*E faecium* (VREf) outbreaks caused by clonal propagation due to interhospital transmission occurred in six general hospitals in Aomori prefecture, Japan.

**Methods:**

The number of patients with VREf was obtained from thirty seven hospitals participating in the local network of Aomori prefecture. Thirteen hospitals performed active screening tests for VRE. Whole genome sequencing analysis was performed.

**Results:**

The total number of cases with VREf amounted to 500 in fourteen hospitals in Aomori from Jan 2018 to April 2021. It took more than three years for the frequency of detection of VRE to return to pre-outbreak levels. The duration and size of outbreaks differed between hospitals according to the countermeasures available at each hospital. Whole genome sequencing analysis indicated *vanA*-type VREf ST1421 for most samples from six hospitals.

**Conclusions:**

This was the first multi-jurisdictional outbreak of VREf sequence type 1421 in Japan. In addition to strict infection control measures, continuous monitoring of VRE detection in local medical regions and smooth and immediate communication among hospitals are required to prevent VREf outbreaks.

## Introduction

Vancomycin (VCM)-resistant enterococci (VRE) are listed by the Centers for Disease Control and Prevention (CDC) of the United States of America (US) as antibiotic-resistant bacteria that present “serious threats” to human health [1]. Spread of VRE is a global concern as a significant cause of healthcare-associated infections [2]. According to data collected by European Centre for Disease Prevention and Control (ECDC) from the European Antimicrobial Resistance Surveillance Network (EARS-Net) and the Central Asian and European Surveillance of Antimicrobial Resistance (CAESAR), significantly increasing trends for vancomycin resistance in *E. faecium* were observed for the period 2016–2020, similar to the previously reported trends for 2015–2019 when the United Kingdom was included [3].

Healthcare-associated VRE infections have been reported in Japanese hospitals since the beginning of this century [4]. As emergence of VRE is uncommon in Japan, even a single case at one hospital is considered an outbreak. A series of *vanA*-positive *E. faecium* outbreaks occurred in Aomori prefecture, at the northern end of Honshu mainland, Japan, between January 2018 and April 2021 that took more than three years to eliminate. All detected VREs in the outbreaks were *E. faecium*. We report the series of VRE outbreaks that were spread by interhospital transmission in Aomori prefecture by *vanA*-type vancomycin-resistant *Enterococcus faecium* (VREf) sequence type 1421.

## Materials and methods

### Data collection

The number of patients sampled for all bacteriological tests in thirty seven hospitals participating in the Aomori Infection Control Network (AICON) was obtained through the Microbiological Information Network Aomori (MINA). AICON is a local infection control network managed by the Infection Control Center at Hirosaki University Hospital. AICON covers approximately 50% of all hospitals including all general hospitals in Aomori prefecture and two laboratory centers. Participating hospitals can share information about infection control through the network. MINA is a bacteriological database run by AICON. The microbiological test results obtained at each hospital were uploaded via an information network and stored in web servers located and managed at the Infection Control Center of Hirosaki University Hospital. The information processing systems for the database was supplied by KD-ICONS (Tokyo, Japan).

The number of patients with VREf detected by active screening was obtained from thirteen hospitals participating in AICON, all of which performed active screening tests for VRE. These data were also included in the database of MINA. Screening examination of stool samples and/or rectal swabs in all patients who had a preceding hospitalization and in new admissions was formally performed between April 2019 and March 2021 according to the protocol of each hospital and the informed consent. Because the expense of screening was burdensome for the hospitals, it was performed at the discretion of each hospital. In all general hospitals and in hospitals at which an outbreak occurred, simultaneous screening examination was carried out for all inpatients at least twice and new admissions during a certain period (for 1–2 years). At other hospitals, a single simultaneous screening for all inpatients and/or screening for at least new high-risk admissions was carried out.

Data regarding the official proportion of VREf on *E. faecium* isolated from all samples in all 47 prefectures of Japan were obtained from Japan Nosocomial Infection Surveillance (JANIS), which is a national surveillance program organized by the Ministry of Health, Labour and Welfare of Japan. Its clinical laboratory division collects information regarding the number of nosocomial infections and antimicrobial-resistant bacteria in Japanese hospitals. As of January 2020, JANIS covered 2,418 hospitals, including > 80% of hospitals with 500 beds or more [5, 6]. JANIS member hospitals are required to submit surveillance data on a regular basis. National data of drug-resistant bacteria in Japan are publicly available on the JANIS website [7]. VRE was defined following the standard provided by the infectious diseases control law (Act on the Prevention of Infectious Diseases and Medical Care for Patients with Infectious Diseases [8]) as *Enterococcus* spp. resistant to vancomycin (VCM) with MIC of ≥ 16 μg/mL in the microdilution method, which is the definition used by JANIS since 2007, and which corresponds to > 4 μg/mL as defined in the international guidelines.

For economic reasons, pulsed-field gel electrophoresis (PFGE) and genome typing could not be applied to all isolates. Accordingly, these analyses were applied to 20 samples from five general hospitals where outbreaks occurred and from Hirosaki University Hospital, which were able to preserve and provide samples during the series of VRE outbreaks in Aomori prefecture.

### Identification of VRE

VRE was identified by the clinical laboratory at each affected hospital according to the CLSI (Clinical and Laboratory Standards Institute). Active screening for VRE was done using stool samples and/or rectal swabs. Prepared fecal samples were inoculated onto VRE selective agar (Becton Dickinson and Company, Franklin Lakes, NJ, USA). Identification of the isolates and drug susceptibility tests were performed by MicroScan WalkAway using Combo panel PC1J (Dade MicroScan, Sacramento, CA, USA). The Biotyper matrix-assisted laser desorption/ionization system (Bruker Daltonics GmbH and Co., Bremen, Germany) in combination with MicroScan WalkAway and PM1J panel was utilized. Several samples isolated in the early phase of outbreaks at each hospital were sent to the Aomori Prefectural Public Health and Environment Center for detection of *vanA* and *vanB* genes using multiplex PCR (polymerase chain reaction).

### Whole genome sequencing

Genomic DNA was extracted with QIAamp DNA purification kit (QIAGEN, Hilden, Germany) according to the manufacturer’s instructions. DNA libraries were prepared for sequencing with Enzymatics 5X WGS reagents (BioStream Co., Ltd, Tokyo, Japan) and then pooled. For Illumina sequencing, paired-end sequencing (2 × 300 bp) was performed using the MiSeq reagent kit v3 on the MiSeq platform. Raw reads were assembled using Shovill v1.0.9 [9] with the default settings. Multilocus sequence typing (MLST) determination was performed using staramr v0.7.2 [10]. For single nucleotide polymorphism (SNP) phylogenetic tree analysis, sequences of each strain were aligned using the Snippy pipeline (v4.4.5) [11]. Reads were mapped to EF_DMG1500501 (a closed annotated Australian genome, Accession No. LT603678). Phylogenetic trees were displayed, annotated, and decorated with FigTree v1.4.4 [12]. All sequence data have been deposited in the DDBJ/EMBL/GenBank databases Sequence Read Archive (DRA) under accession number DRA012428.

## Results

### Outbreak report

#### Setting

**Fig. 1:**
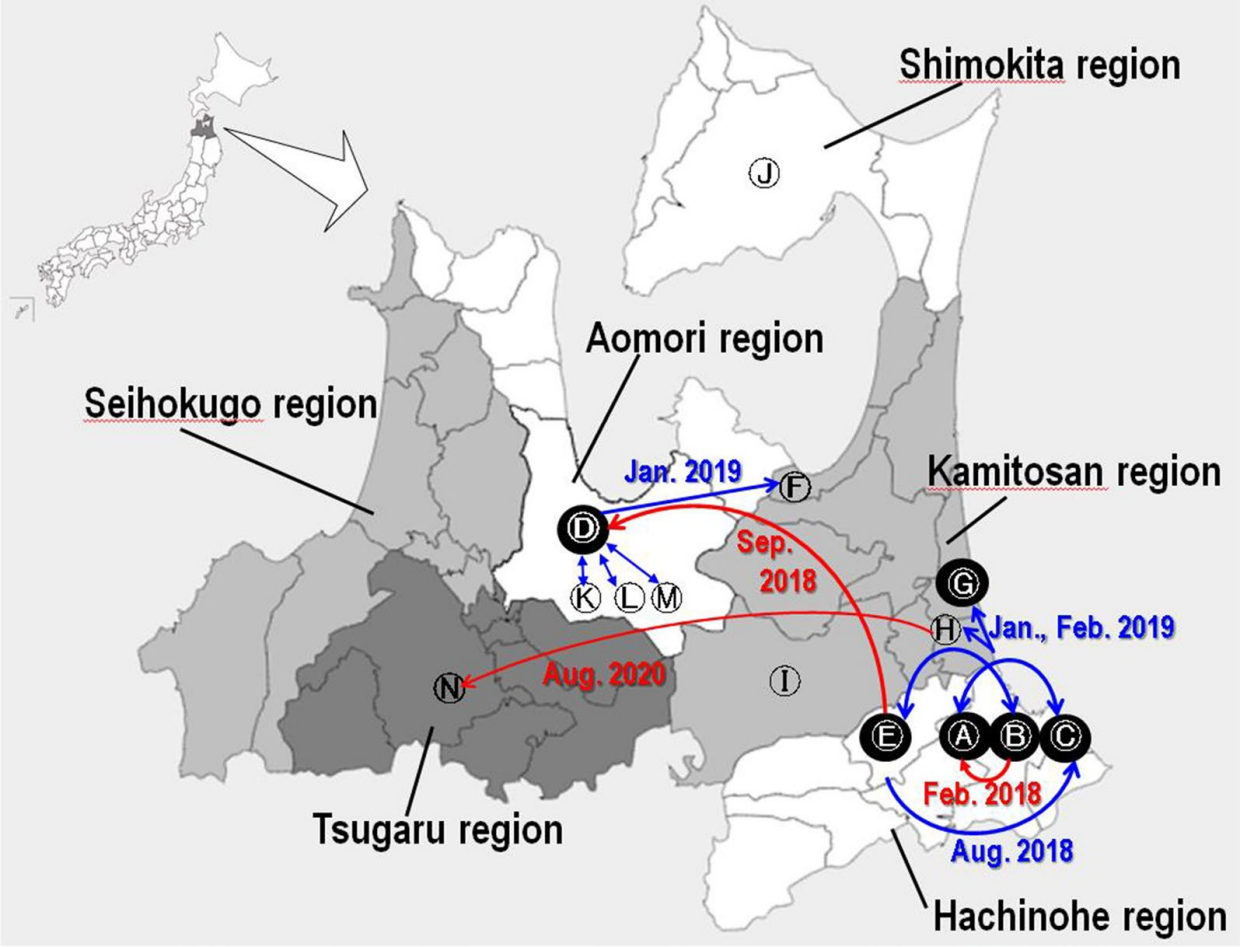
The medical regions in Aomori prefecture and the transmission of VREf. The map shows the locations of the fourteen hospitals in which interhospital VREf transmissions occurred within the six medical regions in Aomori. Red arrows show reference or transfer of VREf carrier between hospitals. Blue arrows show tracking of possible VREf carrier from other hospitals or the area of hospitals in which an outbreak occurred. The described date is the date on which a VREf carrier was detected. Hospitals indicated by the solid black circles had intra-institutional spread of VREf, and required long-lasting strict countermeasures to end the outbreak

**Fig. 2:**
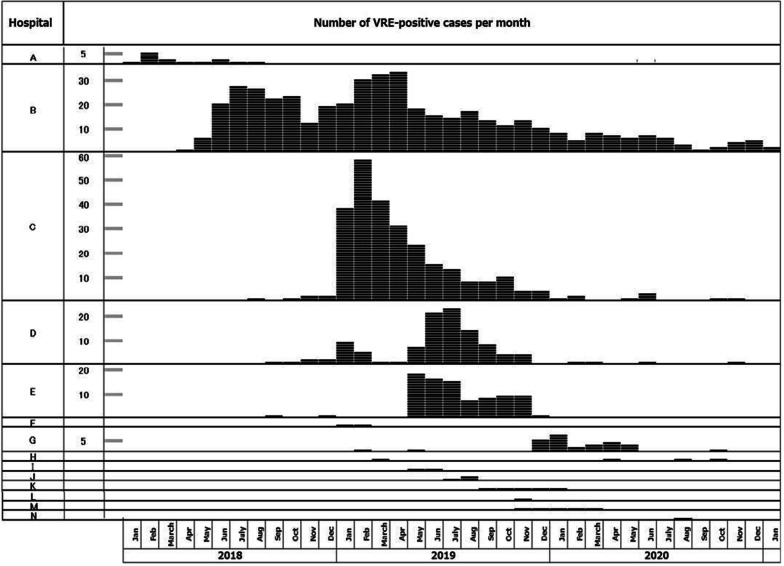
Number of VREf-positive patients per month in all affected hospitals. The monthly number of VREf-positive patients is shown for hospitals A–N. The numbers include new patients and those who repeatedly tested positive for VREf. Hospitals B and C are major tertiary general hospitals in adjacent medical regions

Aomori prefecture has six major medical regions: Hachinohe, Kamitosan, Aomori, Tsugaru, Seihokugo, and Shimokita (Fig. 1). The Hachinohe, Aomori and Tsugaru regions each have a tertiary general hospital with more than 500 beds. There are several medium-sized general hospitals in these six medical regions. During the period from January 2018 to April 2021, outbreaks of VREf which were strongly suspected as interhospital transmission occurred in 14 hospitals in Aomori prefecture, indicated as A–N in chronological order according to the date of the first VREf-positive case identified at each hospital (Fig. 1), and included two tertiary general hospitals. Six hospitals (A, B, C, D, E, and G) had insidious intra-institutional spread of VREf (Fig. 2), and required long-lasting strict countermeasures to end the outbreak. Hospitals B, C, D, and G had infection control teams (ICTs) including full-time staff and belonged to the AICON at the time of onset of the outbreaks, while Hospitals A and E did not belong to AICON and did not have any infection control specialist (Table 2).

Before the outbreak, AICON had regularly held lectures, workshops and meetings about standard precautions and environmental improvements, resistant bacteria, and proper use of antimicrobial. After the onset of the outbreaks, the executive office of AICON discussed the outbreaks with member hospitals and each local public health center in Aomori prefecture, and shared the outbreak data. By the request of the executive office of AICON and local public health centers, an infection control doctor (ICD), infection control nurse (ICN) and infection control microbiological technologist (ICMT) from various AICON member hospitals performed inspections of the hospitals at which outbreaks occurred. They gave guidance regarding intensive observance of standard precautions, cohorting VRE-positive patients (including individualized bathrooms), and recommended stool screening examinations for all inpatients and new admissions, regardless of risk factors.

#### Index case (Hospital A)

Hospital A is a small hospital with < 100 beds in Hachinohe city and has a close relationship with Hospitals B and C in the same medical region (Fig. 1). The first VREf was detected at this hospital in pus from skin in January 2018, followed by a second case three weeks later. The *vanA* gene was detected by PCR in the VREf isolates. Active bacteriological screening of hospitalized patients, as instructed by the health center of Hachinohe region, revealed four additional cases in February 2018. One of those cases had been transferred from Hospital B. The health center shared information with AICON about the VRE outbreak at Hospital A, which was not a member of AICON and did not have any infection control specialist. Strict infection control measures thus had not been undertaken. In March 2018, an emergent external inspection was carried out at Hospital A by the public health center of Hachinohe region. Genetic and PFGE analyses suggested healthcare-associated transmission of *vanA*-type VREf. A total of 15 cases were identified at this hospital during the eight-month period of the outbreak.

#### Hospital B

Following the outbreak at hospital A, VREf outbreaks occurred in five general hospitals (B, C, D, E, and G) from 2018 to 2020 (Fig. 2). Hospital B, a general hospital with > 400 beds, received the report that one patient transferred from Hospital B to Hospital A was a VREf carrier from the health center of Hachinohe region in February 2018 (Fig. 1). After that, five VREf-positive patients were identified in a medical ward in May 2018 following the identification of one patient in April 2018. VREf spread to five other wards in the next few months despite countermeasures that included patient cohorting, active surveillance, and education of medical staff. A second outbreak then occurred in December 2018, during which all inpatients were screened monthly and VREf-positive patients were cared for in a newly prepared isolation ward. Four external inspections were carried out during this long-standing outbreak (June 2018, January 2019, February 2019, and March 2019) by different teams of specialists sent from among AICON, the Japan Red Cross Society, Iwate University Hospital, and the National Institute of Infectious Diseases. Active surveillance of all new admissions detected very few VRE-positive patients, indicating that there was no spread of VREf within the community. It took 33 months for hospital B to end the outbreak.

#### Hospital C

Hospital C, a tertiary general hospital in the same city as Hospitals A and B, noticed the insidious intra-institutional spread of VREf in the two adjacent hospitals at that time based on information from AICON and the public health center in the region. The laboratory department therefore started actively trying to identify VRE as much as possible, detecting the first case of VREf in August 2018. That case, involving a patient receiving chemotherapy for otolaryngological cancer, was identified from the emergency department. The patient was living near Hospital E in a town next to Hachinohe city (Fig. 1). Despite countermeasures such as isolation and strict standard precautions, active screening in the emergency department revealed an abrupt increase in positive cases between December 2018 and January 2019, and a patient with severe burns was infected with VREf during this period. It was thought that meticulous wound care and handling of wet wound coverings contaminated with VREf would minimize the risk of spread; however, screening of all patients revealed additional VREf-positive cases in other wards. Scheduled hospitalizations and surgeries were postponed and the hospital accepted only emergent cases. External inspection was carried out by team of specialists sent by Hirosaki University Hospital, a member of AICON. An isolation ward was prepared as soon as the spread of VREf within the hospital was confirmed. Monthly screening of all inpatients was repeated between February 2019 and December 2019. The spread of VREf was brought under control after February 2019, and the number of VREf-positive patients decreased gradually thereafter.

#### Hospital D

Hospital D is a tertiary general hospital in Aomori city far from Hospitals A, B, C, and E in Hachinohe region. Hospital D also noticed the insidious intra-institutional spread of VREf in Hachinohe region at that time based on information from AICON. One VREf-positive patient, who had been transferred from Hospital E, was found in September 2018 (Fig. 1), followed by two more patients in November 2018. An additional six cases were found in screening of patients hospitalized on the same floor in December 2018. The first wave of the VREf outbreak was controlled by regular countermeasures such as strict standard precautions, patient isolation, and active VRE screening of new patients at hospitalization and at every month after hospitalization in the ward. After the curve of the epidemic showed a nadir in February 2019, a second outbreak occurred in May 2019, during which countermeasures for VRE-outbreak in the hospital were evaluated by specialists from the local government, Aomori City Health Center, AMR Clinical Reference Center at the National Center for Global Health and Medicine, and AICON, during an external inspection in December 2018. Monthly screening of all inpatients was repeated from May 2019. Active screening for VRE during the period from September 2019 to July 2020 revealed that 12/49 (24.5%) VRE-positive cases were already colonized with VREf at the time of admission. VREf-positive patients at admission suggested the spread of VRE within the Aomori region. The number of VREf-positive patients gradually decreased after August 2019, and returned to zero in December 2019.

#### Hospital E

Hospital E is a small general hospital in the Hachinohe region, the same region as Hospitals A, B, and C. Patients are often transferred from and referred to general hospitals B and C. Hospital E did not belong to AICON and noticed that a VREf carrier was in Hospital E in September 2018 based on a report from Hospital D (Fig. 1). However, active VRE screening was not performed until May 2019 in accordance with hospital policy. Because of the VREf outbreak at Hospital B, active VRE screening began in May 2019 according to strong recommendations by AICON and the public health center in Hachinohe region. Six VREf-positive inpatients were found at the first screening. Monthly screening of all inpatients was introduced after the first external inspection by infection control specialists arranged through AICON. Follow-up external inspection was carried out in late November 2019. Infection control activities such as standard precautions, zoning, environmental hygiene, and education were evaluated and encouraged. The number of VREf-positive patients returned to zero in January 2020.

#### Hospital G

Hospital G is a medium-sized general hospital in Kamitosan region, situated slightly separate from Hachinohe city. Patients are sometimes transferred from and referred from general hospitals B, C, and D (Fig. 1). The first VREf-positive patient was found in February 2019, and cases with VREf were found sporadically during periods of VREf outbreaks at other hospitals. The local health center of Kamitosan detected sporadic cases of VREf-positive patients in hospitals in their area of responsibility. Following the recommendation of the local health center, the first screening for VRE was carried out for hospitalized patients in December 2019, and found five VREf-positive patients. In addition to the reinforcement of routine infection control activities, VRE screening of patients at hospitalization was started after the first screening. The outbreak ended within six months.

#### Other hospitals

From 2019, AICON recommended that participating hospitals should screen for VRE at hospitalization, which was conducted at a total of 13 hospitals: 12 general hospitals and 1 special hospital. A small number of VREf-positive patients were detected by this screening, although it was difficult to discriminate between minimal intrahospital transmission and individual VRE carriages. No new VRE-positive cases were detected after May 2021.

### Epidemiological summary

The total number of cases with VREf amounted to 500 in 14 hospitals in Aomori prefecture from Jan 2018 to April 2021. Table 1 shows the number of all bacteriological tests performed in all AICON member hospitals in Aomori prefecture (MINA data), and the numbers of new VREf-positive patients and positivity rates detected by active screening at the 13 hospitals. The number of patients tested for all bacteriological cultures showed a remarkable increase during the VRE outbreak period. The total number in 2019 was 48.4% greater than that in 2017.

**Table 1:** Number and rate of VREf in all samples and blood culture in AICON (MINA data), and number and rate of VREf from active screening in 13 hospitals

	2017–2018		2018–2019		2019- 2020		2020–2021		2021-
	Apr-Sep	Oct-Mar	Apr-Sep	Oct-Mar	Apr-Sep	Oct-Mar	Apr-Sep	Oct-Mar	Apr-Sep
All samples (Cases)	30,783	29,862	31,929	36,268	44,540	45,432	40,017	39,317	35,974
*E. faecium* (Cases)	257	218	267	264	256	295	266	277	251
(The percentage of *E. faecium* in all samples)	(0.83%)	(0.73%)	(0.84%)	(0.73%)	(0.57%)	(0.65%)	(0.66%)	(0.70%)	(0.70%)
VREf data (Cases)	0	1	54	178	169	55	28	14	1
VREf */* all samples	0.00%	0.00%	0.17%	0.49%	0.38%	0.12%	0.07%	0.04%	0.00%
**VREf */ E. faecium***	0.00%	0.46%	**20.22%**	**67.42%**	**66.02%**	**18.64%**	**10.53%**	**5.05%**	0.40%
Blood culture (Cases)	8585	8606	8668	8499	9667	9118	8980	10,513	8847
*E. faecium* in blood culture (Cases)	38	33	42	34	46	65	39	60	58
(The percentage of *E. faecium* in blood culture)	(0.44%)	(0.38%)	(0.48%)	(0.40%)	(0.48%)	(0.71%)	(0.43%)	(0.57%)	(0.66%)
VREf in blood culture (Cases)	0	0	1	10	8	2	0	0	0
VREf */* blood culture	0.00%	0.00%	0.01%	0.12%	0.08%	0.02%	0.00%	0.00%	0.00%
**VREf */ E. faecium* in blood culture**	0.00%	0.00%	**2.38%**	**29.41%**	**17.39%**	**3.08%**	0.00%	0.00%	0.00%
Active screenig for VREf in 13 hospitals (Cases)					20,676	15,636	10,598	5122	
Simultaneous screening for all inpatients (Cases)					5478	3087	1397	153	
Screening for new admissions (Cases)					15,198	12,549	9201	4969	
New VREf from active screening (Cases)					23	12	2	0	
**New VREf */* active screening**					**0.11%**	**0.08%**	**0.02%**	0.00%	

The proportion of VREf on *E. faecium* in all bacteriological tests increased to a maximum of 67.4% and the rate of VREf on all samples in hospitals belong to AICON increased to a maximum of 0.49% between October 2018 and March 2019. The proportion of VREf on *E. faecium* in blood culture increased to a maximum of 29.4% and the rate of VREf on blood cultures increased to a maximum of 0.12% between October 2018 and March 2019.

In active screening at the 13 hospitals, data are limited as they were obtained only after April 2019. There were 37 VREf-positive cases in 52,032 screening tests (positive rate, 0.07%) in the two years from April 2019 to March 2021. The rate of VREf on active screening samples was highest (0.11%) in the six months between April and September 2019 (Table 1).

### Changes in the proportion of VREf on *E. faecium* in all 47 prefectures of Japan

**Fig. 3:**
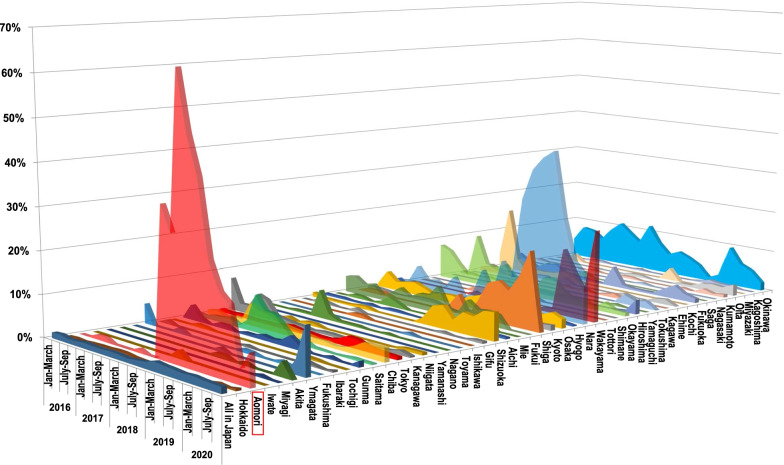
Changes in the proportion of VREf on *E. faecium* all 47 prefectures of Japan. Outbreaks at multiple hospitals in the same period caused a sharp peak in the proportion of VREf. The remarkable size of the area under the curve representing Aomori prefecture indicates the epidemiological importance of the series of VREf outbreaks in this prefecture

Figure 3 shows changes in the proportion of VREf on *E. faecium* in all 47 prefectures of Japan from January 2016 to December 2020, obtained from the JANIS surveillance data (number of cases) including all samples. These outbreaks at multiple hospitals in the same period caused a sharp peak in the proportion of VREf. The remarkable size of the area under the curve representing Aomori prefecture suggests the epidemiological importance of the series of VREf outbreaks in this prefecture.

### Analysis of VRE strains using PFGE

**Fig. 4:**
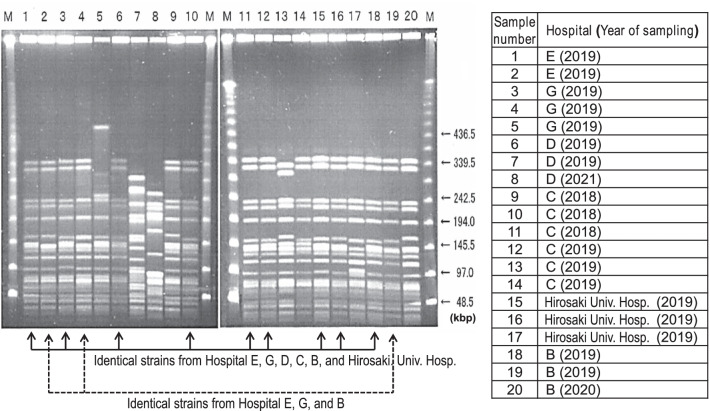
PFGE results of 20 VREf strains isolated from five general hospitals and Hirosaki University Hospital during the VRE outbreaks. Sample numbers are the same as in the right list. kbp: kilobase pairs, M: DNA size marker (lambda ladder)

The right in Fig. 4 lists six source hospitals (Hospitals B, C, D, E, and G and Hirosaki University Hospital) and sampling year for 20 isolates that were tested by PFGE analysis. VRE strains showed identical or very close patterns in PFGE analysis. Lanes 1, 3, 6, 10, 11, 12, 15, 16, and 18 were judged as identical strains. Lanes 2, 4, and 19 were also identical. The former and the latter were judged as closely related with more than 85% similarity, which indicates interhospital clonal spread of VRE in this series of outbreaks.

### SNPs phylogenetic tree analysis

**Fig. 5:**
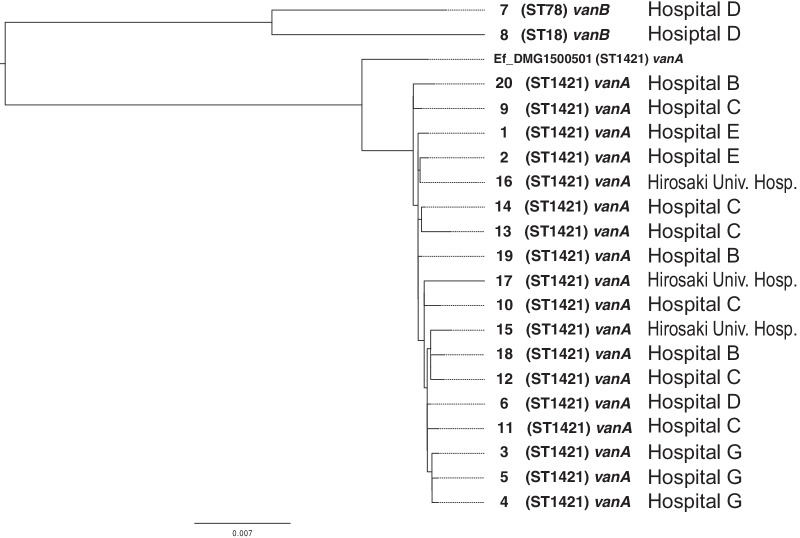
Core genome SNP phylogenetic tree of 20 isolates from five general hospitals and Hirosaki University Hospital during the VRE outbreaks. Sample numbers are the same as listed in Fig. 4. Reference genome: Ef_DMG1500501

Figure 5 shows the phylogenetic tree based on the core genome SNPs of the same 20 isolates. Whole genome sequencing analysis indicated *vanA*-type VREf ST1421 for most samples except samples 7 and 8 from hospital D. These were identified as other *vanB*-type strains, although sample 6, also from Hospital D, was identified as *vanA*-type VREf ST1421, the same as the strains from Hospitals B, C, E, and G.

### Countermeasures to VRE in six hospitals (A, B, C, D, E, and G)

Table 2 shows the situation of infection control in 2018 and the countermeasures against VRE implemented in the 6 hospitals with intra-institutional spread of VRE. Countermeasures 1–10 in each hospital are discussed later.

**Table 2:** Situation of infection control and countermeasures against VRE in six hospitals with intra-institutional spread of VRE

	Hospital A (< 100 beds)	Hospital B (> 400 beds)	Hospital C (> 500 beds)	Hospital D (> 500 beds)	Hospital E (< 200 beds)	Hospital G (< 300 beds)
(1) ICT or Task force for VRE, and rapid reporting to ICT	No	Yes	Yes	Yes	No	No
(2) Rapid laboratory identification of VRE	Delayed	Yes	Yes	Yes	Delayed	Delayed
(3) Rapid and repeated hospital-wide screenings	Only once	Yes	Yes	Yes	Very delayed	Delayed
(4) Cohorting patients with dedicated staff into sections: "VRE patients"; "Contact patients"; and "VRE-free patients"	Only VRE section	Yes	Yes	Only VRE section	Yes, but delayed	Yes, but not dedicated staff
(5) Stopping transfers of VRE patients and contact patients to other units or to any other hospitals	Only VRE patient	Yes, but delayed	Yes	Yes	Yes, but after once negative	Yes
(6) Extended and maintained screening of contact patients already discharged or transferred until the outbreak is controlled	No	Yes	No	Yes	Yes	Yes
(7) Flagging of medical records for identifying discharged VRE patients and contact patients in case of readmission	Only VRE patient	Only VRE patient	Only VRE patient	Only VRE patient	Only VRE patient	Only VRE patient
(8) Environmental screening and increased cleaning		Yes	Yes	Yes	Yes	Yes
(9) Antimicrobial stewardship	No	Insufficient	Yes	Insufficient	No	No
(10) Information sharing using a local network for infection control (Belong to AICON in 2018)	No	Yes	Yes	Yes	No	Yes

## Discussion

VRE had spread worldwide by the year 2000 [13]; however, the frequency of detection of VRE was still low in Japan as of 2019 [14]. Using the ECDC surveillance data as a measure of bloodstream infection (BSI) incidence could underestimate the true value in countries with low blood culture frequency [15]. The data show that resistance to vancomycin in *E. faecium* varied substantially among countries in Europe and Central Asia. In 2020, resistance percentages < 1% were reported by 7/38 (18%) countries reporting data on VREf, whereas percentages ≥ 25% were found in 13/38 (34%), and 4/38 (11%) reported resistance percentages ≥ 50% [3]. Unfortunately, JANIS does not hold data of the resistance percentage of *E. faecium* in blood culture. However, the resistance percentage in all samples in Japan remained at a level around 1.0–1.5% except the period from January to July in 2019, when the outbreaks and active screenings in Aomori prefecture were at peak levels (Fig. 3). The resistance percentage of *E. faecium* in blood culture in Aomori was 0% for several years until March 2018, but was 29.4% for the six months from October 2018 to March 2019 and 22.5% for the year from October 2018 (Table 1). These rates are exceptionally high for Japan.

It is not easy to prevent VRE spread without active screening because patients infected (colonized) with VRE are usually asymptomatic. By the time an index VRE carrier is found by chance, several asymptomatic patients in proximity to the index case can be found by spot surveillance, as was the case in hospitals in the present series. It takes months for colonized patients to be free from VRE [16]. Furthermore, colonized patients show no obvious signs of infection but can spread VRE through person-to-person contact during hospitalization.

Christiansen et al. [17] reported that one hundred sixty-nine cases in 23 wards were colonized with a single strain of VREf in a major Australian hospital. The following interventions were introduced: (1) Formation of a VRE executive group; (2) Rapid laboratory identification; (3) Screening of all inpatients with isolation of carriers and cohorting of contacts; (4) Environmental screening and increased cleaning; (5) Electronic flagging of the medical records of contacts; and (6) Antibiotic restrictions. Using these interventions, the outbreak was terminated within only 3 months, but at a cost of 2.7 million Australian dollars (1.9 million US dollars). In a 1600-bed hospital in Singapore, eradication of a hospital-wide outbreak comprising 151 cases required a coordinated strategy comprising: (1) Formation of a VRE task force; (2) Hospital-wide screening; (3) Isolation of carriers; (4) Physical segregation of contacts; (5) Surveillance of high-risk groups; (6) Increased cleaning; (7) Electronic tagging of VRE status; and (8) Education and audits [18]. Furthermore, Fournier et al. reported that 45 repeat outbreaks of VREf occurred, comprising 533 cases, between 2004 and 2010 in a 23,000-bed multi-hospital institution in France [19]. During that period, a multidrug-resistant bacteria control program was implemented, including the following measures: (1) Rapid reporting to ICT; (2) Stopping transfers of cases and contact patients to other units or to any other hospitals; (3) Particular attention to daily cleaning of VRE patient environments; (4) Extended screening of contact patients already discharged or transferred from the involved unit; (5) Maintained screening of all contact cases until the outbreak was controlled; (6) Identification of discharged VRE patients and contact patients in case of readmission; and (7) Cohorting patients in three distinct areas with dedicated nursing staff: “VRE patients” section, “Contact patients” section and “VRE-free patients” section for newly admitted patients. The number of cases per outbreak was significantly lower after implementation of this program.

These strategies can be summarized as follows.

 < Strategies for infection prevention and control of VRE infections > 

1. Formation of a VRE executive ICT and rapid reporting to ICT
2. Rapid laboratory identification of VRE
3. Rapid and repeated hospital-wide screenings
4. Cohorting patients with dedicated staff into sections: “VRE patients”; “Contact patients”; and “VRE-free patients”
5. Stopping transfers of VRE patients and contact patients to other units or to any other hospitals
6. Extended and maintained screening of contact patients already discharged or transferred until the outbreak is controlled
7. Flagging of medical records for identifying discharged VRE patients and contact patients in case of readmission
8. Environmental screening and increased cleaning.
9. Antimicrobial stewardship.

In addition to the above, the common understanding of the need for screening, patient selection, and evaluation of the clinical and economic benefits based on the frequency of detection of VRE should be shared by cooperating hospitals within medical regions. Interhospital patient transfer is commonly required to improve patient management; however, transferring patients can promote interhospital transfer of multidrug-resistant organisms (MDROs). Therefore, “Information sharing using a local network for infection control” could be added as the 10th strategy for preventing MDROs such as VRE.

We investigated these 10 items in the six hospitals that had experienced intra-institutional spread of VRE (Table 2). Countermeasures to “contact patients” were totally insufficient, and identification of VRE and hospital-wide screening were delayed, particularly in two small hospitals (A and E) that did not belong to AICON at that time. Hospitals A, B, C, and E are in the same medical region and have close relationships with each other (Fig. 1). Interhospital transmission of VREf is strongly suspected to have occurred from the above reasons. It is difficult to define an interhospital transmission, but comparison between the consistently low frequency of detection of VRE in Japan (Fig. 3) and the remarkably high proportion of VRE on *E. faecium* isolated from blood culture and in all samples from Aomori prefecture (Table 1), which is very uncommon in Japan, indicate interhospital transmission. Interhospital VRE transmissions have been reported in Australia [20], Taiwan [21], and Kyoto (Japan) [22]. If VRE is detected in a hospital, the information should be shared by other hospitals in the medical region to prevent interhospital transmission. In their systemic review, Urlich et al. (2017) [23] reported that the duration of VRE outbreak in hematology and oncology departments ranged from 1 to 36 months, with a mean value of 11 months. Strong countermeasures including active VRE screening are necessary to shorten the duration of VRE outbreaks [24–26].

Increases in medical costs due to spread of VRE is a matter of concern in countries such as the United States [27], Germany [28], France [29], China [30] and Canada [31]. The cost-saving effect has been shown in a detailed analysis of the costs associated with implementation of a strict policy for controlling spread during outbreaks, including active screening for highly resistant microorganisms [32]. Hospital administrators and infection control specialists need to discuss the costs and benefits of VRE screening to achieve efficient and effective countermeasures based on the medical conditions in each area [33].

PFGE and whole genome sequencing analysis clearly indicated that the multi-jurisdictional outbreaks were caused by *vanA*-type VREf ST1421, whereas 2 of 20 isolates showed different patterns of PFGE and were identified as other *vanB*-type strains by whole genome sequencing. The *vanA* and *vanB* types could generally be distinguished by susceptibility, but confirmation by genetic analysis was useful. The ST1421 clone lacks the *pstS* housekeeping gene of the MLST allele [34]. *pstS*-null VREf clones have recently emerged worldwide and regional spread has been reported in numerous countries, including Australia [34], the UK [35], Scotland [36], Denmark [37] and Korea [38]. To the best of our knowledge, this is the first report of a VRE outbreak by *pstS*-null VREf ST1421 in Japan.

### Study limitation

Data assessed in the present study were obtained from the MINA database, which targeted all clinical samples from approximately 50% of all hospitals (including all general hospitals) and laboratory centers belonging to AICON in Aomori prefecture. However, data from small sized hospitals and clinics which do not make it their routine work to collect bacteriological samples were not included. Therefore, it is possible that some VRE carriers could have been missed.

Furthermore, because our study was limited regarding clinical data from VRE-positive patients, we could not investigate the burden of the infectious disease as compared to asymptomatic carriage. That would have provided more useful information, as symptomatic/infected individuals would likely accumulate higher associated medical costs.

## Conclusion

Interhospital transmission of VREf ST1421 occurred in Aomori prefecture despite a prefecture-wide infection control network that provides data of microbiological test results uploaded from 37 hospitals in the prefecture. This was the first multi-jurisdictional outbreak of VREf sequence type 1421 in Japan. In addition to strict infection control measures and a system of continuous monitoring of MDROs, sustained interest in the frequency of detection of VRE in local medical regions and smooth and immediate communication among hospitals are required to prevent VRE outbreaks.

## Data Availability

The data that support the findings of this study are available from the corresponding author, Saito N, upon reasonable request.

## Abbreviations

VREf: Vancomycin-resistant *Enterococcus* *faecium*
CDC: Centers for disease control and prevention
ECDC: European centre for disease prevention and control
EARS-Net: European antimicrobial resistance surveillance network
CAESAR: Central Asian and European surveillance of antimicrobial resistance
AICON: Aomori infection control network
MINA: Microbiological information network Aomori
JANIS: Japan nosocomial infection surveillance
MIC: Minimum inhibitory concentration
PFGE: Pulsed-field gel electrophoresis
MLST: Multilocus sequence typing
SNP: Single nucleotide polymorphism
ICT: Infection control team
AMR: Antimicrobial resistance
MDROs: Multidrug-resistant organisms
